# Preschoolers’ cognitive flexibility and emotion understanding: a developmental perspective

**DOI:** 10.3389/fpsyg.2024.1280739

**Published:** 2024-02-08

**Authors:** Li Mengxia

**Affiliations:** Faculty of Management of the Southern Federal University, Rostov-on-Don, Russia

**Keywords:** preschoolers, cognitive flexibility, facial emotion recognition, situational emotion understanding, developmental perspective

## Abstract

**Introduction:**

Cognitive flexibility is the ability to adapt to changing tasks or problems, while emotion understanding is the ability to interpret emotional cues and information in different contexts. Both abilities are crucial for preschoolers’ socialization.

**Methods:**

This study selected 532 preschool children aged 3–6 years from two kindergartens in a central province of China. The Dimensional Change Card Sorting (DCCS) task and emotion understanding tasks were used to investigate the developmental characteristics of cognitive flexibility, emotion understanding abilities, and their relationship.

**Results:**

The results showed: (1) For cognitive flexibility, children older than 5 years scored significantly higher than younger children, and girls scored higher than boys. (2) For facial emotion recognition: (i) Children’s recognition scores for happy, sad, and angry expressions were significantly higher than fear; children could accurately recognize happy, sad, and angry emotions by age 3, while fear recognition developed rapidly after age 5; (ii) Girls scored higher in recognizing fearful faces than boys. (3) For situational emotion understanding: (i) Children’s development followed the hierarchical order of external, desire, clue, and belief-based understanding. Situational and desire-based understanding already reached high levels by age 3, while clue and belief-based understanding developed quickly after age 5; (ii) Girls scored higher than boys in belief-based emotion understanding. (4) Cognitive flexibility significantly predicted children’s facial emotion recognition, external and desire-based emotion understanding.

**Discussion:**

Parents and teachers should cultivate children’s cognitive flexibility and provide personalized support. They should also fully grasp the characteristics of children’s emotion understanding development, systematically nurture their emotion understanding abilities, and leverage cognitive flexibility training to improve their emotion understanding.

## 1 Introduction

The preschool period refers to the stage of early childhood education that precedes formal schooling and is typically intended for children between the ages of three and six. During this time, significant advancements in physical development occur, while crucial psychological functions such as executive function and emotional abilities undergo critical stages of development (Hongwanishkul et al., 2016). Research has found that preschoolers’ executive function and emotional abilities are not only significantly correlated with current motor coordination, teacher-student relationships, and peer relationships (Brown and Dunn, 1996; Xu and Guo, 2004; Garner and Waajid, 2008; Piek et al., 2008; Holmes et al., 2016; Fang et al., 2017; McKinnon and Blair, 2018), but can also predict academic performance, prosocial behavior, and problem behaviors in the school-age period and even later in life (Eisenberg et al., 1997; Trentacosta and Izard, 2007; Pavarini et al., 2011; Sulik et al., 2015; O’Toole et al., 2017; Morgan et al., 2019). However, current research on executive function and emotional abilities in preschoolers has mainly focused on inhibitory control and emotion regulation. There is still a lack of empirical studies investigating the developmental characteristics of cognitive flexibility and emotion understanding in preschoolers, as well as the relationship between the two.

Cognitive flexibility refers to the ability of young children to inhibit dominant rules and shift from one response set to another cognitive rule that can adapt to changes (Ionescu, 2012). Research conducted by Blakey et al. (2016) demonstrated that two-year-old children do not possess cognitive flexibility. Frye et al. (1995) discovered that three-year-olds have difficulties with rule shifting, while four-year-olds performed better on card sorting tasks compared to three-year-olds. Vitiello et al. (2016) believed that age five is the period when cognitive flexibility develops most rapidly in young children. However, there is still a lack of research investigating the developmental trajectory of cognitive flexibility across the entire preschool period from ages 3–6 years. There is also no consensus on gender differences in cognitive flexibility among preschoolers (Patwardhan et al., 2021). Further research is warranted to comprehensively understand the intricate relationship between gender and cognitive flexibility in this particular age group.

Emotion understanding refers to an individual’s social cognitive ability to understand the relationship between emotions, the situations that evoke them, and the behaviors they manifest (Pons et al., 2004). Currently, research on emotion understanding in preschoolers has mainly focused on facial expression recognition, emotion understanding based on external factors, desires, beliefs, cues (memory). Wang et al. (2010) study indicated that children can recognize facial expressions and understand external causes of emotions by around 3 years of age. Jiang and Tan (2018) research showed that children can understand desire-based emotions by around 4 years old. Mou and Chen (2006) found that children cannot understand belief-based emotions until around five and a half years old. Yang and Hu (2003) discovered that six-year-old children can utilize clue information to facilitate emotion understanding. While the above studies answered questions about specific stages of emotion understanding abilities, there is still a lack of research investigating the longitudinal developmental trajectories of different levels of emotion understanding abilities. Similarly, there is also no consensus on gender differences in emotion understanding. For example, Bosacki and Moore (2004) study showed that girls are better at understanding complex emotions compared to boys. Yao et al. (2004) research also found that girls had higher abilities in inferring others’ emotional states compared to same-age boys. However, Mou and Chen (2006) study showed no significant gender differences in children’s emotion understanding.

Cognitive flexibility and emotion understanding belong to the two domains of cognition and emotion, respectively. However, these two domains do not exist independently. Given the integrality of the human brain and its functions, cognitive and emotional processes are complex interactive processes involving many physiological and psychological levels. Although the relationship between cognitive flexibility and emotion understanding in young children is still unclear, there seems to be some connection between the two conceptually and based on empirical evidence. On one hand, conceptually, cognitive flexibility is defined as the higher cognitive ability to consider multiple ideas and flexibly shift cognitive schemas to change habitual responses when environmental contingencies change. Emotion understanding is defined as children’s ability to understand the relationship between changes in situations and the corresponding behaviors. Both focus on the accuracy of children’s reactions to changing situations, hence there is some conceptual overlap. On the other hand, empirical studies have also examined this issue. Silkenbeumer et al. (2016) found that the development of children’s emotion understanding requires cognitive flexibility to constrain rigid mindsets and coordinate internal and external cues to adapt to the environment. Understanding more complex emotions requires children to flexibly deal with misleading contextual or emotional cues, which helps them inhibit dominant responses and reconfigure new comprehension schemes. Therefore, cognitive flexibility may be a necessary condition for children’s emotion understanding.

In summary, previous studies on the developmental characteristics of cognitive flexibility and emotion understanding in preschoolers have been inadequate. Additionally, the inconsistent findings from studies with small sample sizes make the relationship between cognitive flexibility and emotion understanding in young children unclear. Therefore, this study recruited a relatively large sample of 532 children aged 3–6 years to systematically investigate the characteristics of cognitive flexibility and emotion understanding development in preschoolers, as well as the relationship between the two. The study aims to address the following research questions: 1. What are the developmental characteristics of cognitive flexibility in preschoolers aged 3–6 years? 2. How does cognitive flexibility develop across the entire preschool period? 3. Are there any gender differences in cognitive flexibility among preschoolers? 4. What are the developmental characteristics of emotion understanding in preschoolers aged 3–6 years? 5. What are the longitudinal developmental trajectories of different levels of emotion understanding abilities? 6. Is there a consensus on gender differences in emotion understanding among preschoolers? 7. What is the relationship between cognitive flexibility and emotion understanding in preschoolers? Exploring these questions holds important practical significance for understanding the development of cognitive flexibility and emotion understanding in young children, guiding school readiness, and early assessment and intervention of related problem behaviors.

## 2 Methods

### 2.1 Participants

Using convenient sampling, 532 preschool children from two representative kindergartens in Kaifeng, Henan Province were recruited as valid participants. The initial dataset consisted of 552 children. However, after careful screening and evaluation, 20 children were excluded from the final analysis due to incomplete or inconsistent data. These kindergartens were known for their diverse student population and were considered to be typical of the preschool education system in the region. The recruitment process involved several steps to ensure the selection of eligible participants. First, we obtained permission from the relevant authorities and the management of the selected kindergartens to conduct the study. Subsequently, we communicated with the preschool teachers and parents to explain the purpose and procedures of the study. Parents were provided with written consent forms and were requested to provide their consent for their child’s participation. Informed consent was obtained from both the preschool teachers and parents before any data collection activities took place. Considering the rapid development of emotion understanding during this stage, to more closely observe the developmental trajectories of cognitive flexibility and emotion understanding, we grouped children based on age in months (6 months per group) (Zhang et al., 2015). Specifically, there were 49 children aged 3–3.5 years (37–42 months) (20 boys, 29 girls), 69 children aged 3.5–4 years (43–48 months) (35 boys, 34 girls), 121 children aged 4–4.5 years (49–54 months) (59 boys, 62 girls), 89 children aged 4.5–5 years (55–60 months) (50 boys, 39 girls), 93 children aged 5–5.5 years (61–66 months) (55 boys, 38 girls), 76 children aged 5.5–6 years (67–72 months) (43 boys, 33 girls), and 35 children aged 6–6.5 years (73–78 months) (21 boys, 14 girls).

### 2.2 Research tools

#### 2.2.1 Cognitive flexibility

**Figure 1:**
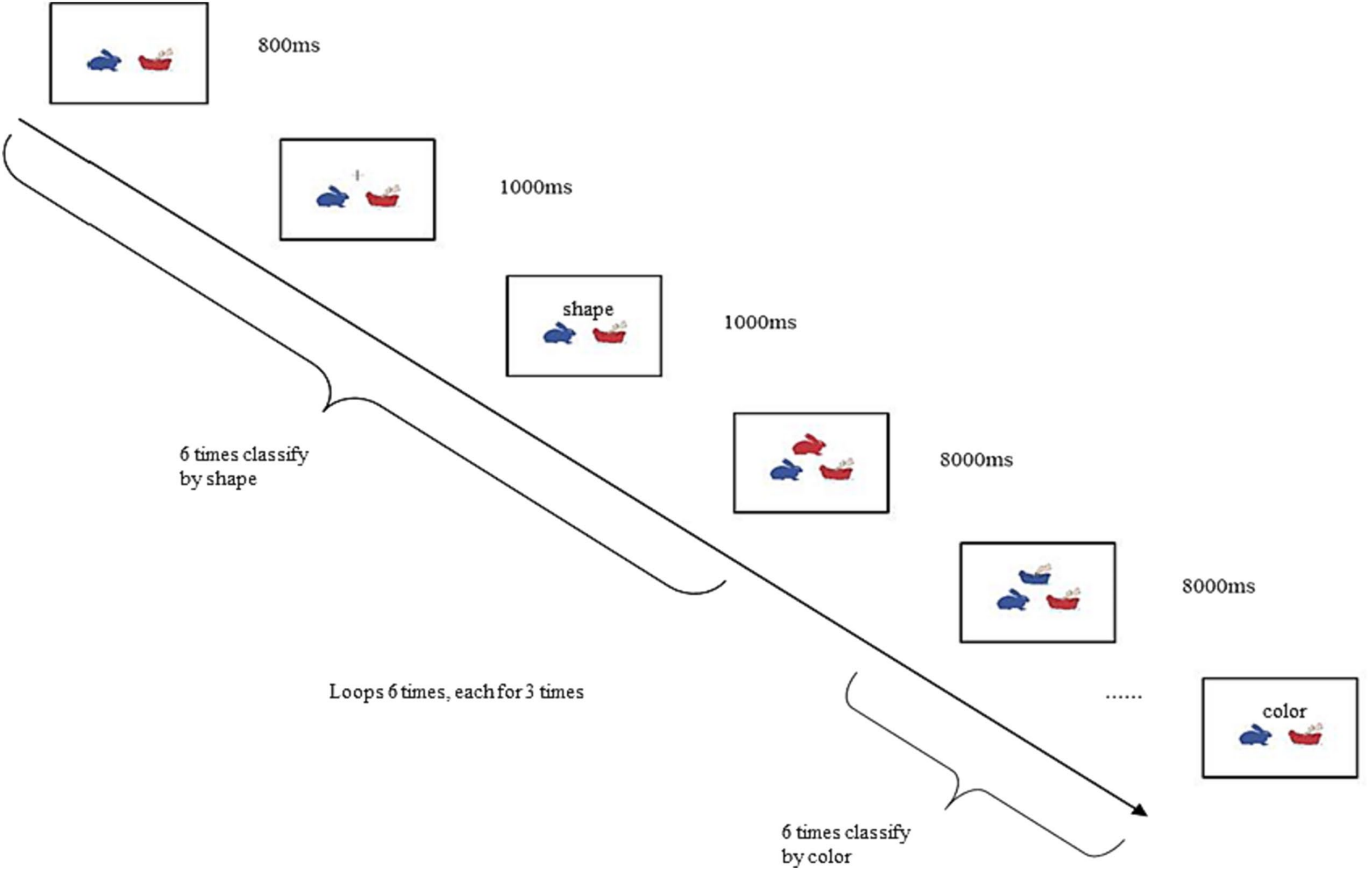
Experimental procedure for the dimensional change card sort (DCCS) task.

Cognitive flexibility was measured using the Dimensional Change Card Sort (DCCS) task (Li and Wu, 2006). First, a card with a blue rabbit and a red boat was displayed on the computer screen. Then, a color or shape cue appeared above the card, indicating the task requirements for the child. Next, a red rabbit or blue boat was presented above the card. In the color task, the child was required to classify the image above as either a blue rabbit (by pressing the left mouse button) or a red boat (by pressing the right mouse button) based on the color cue. In the shape task, the child was required to classify the image above as either a blue rabbit (by pressing the left mouse button) or a red boat (by pressing the right mouse button) based on the shape cue. The response was assisted by the experimenter. After the child had completed six trials based on the shape dimension, the child was then required to complete six trials based on the color dimension, followed by another six trials based on the shape dimension. The procedure for presenting a single stimulus was as follows: the background interface was presented for 800 ms, followed by a “+” fixation point for 1,000 ms, the stimulus image was presented for 1,000 ms, and the response interface was presented for 8,000 ms. The DCCS task consisted of a total of 36 trials, and the specific experimental procedure is shown in Figure 1.

#### 2.2.2 Facial expression recognition

**Figure 2:**
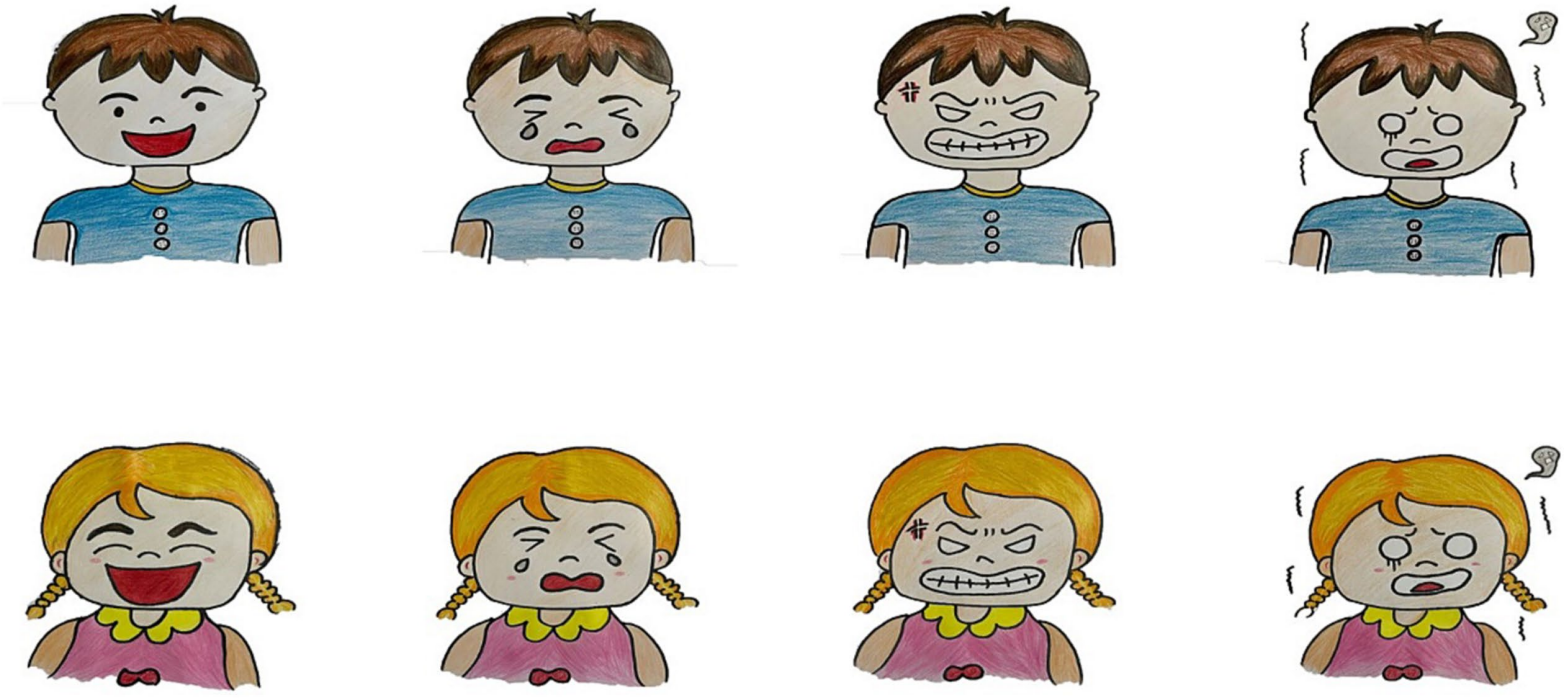
Experimental materials for the facial expression recognition task (from left to right: happy, sad, angry, and fearful).

Facial expression recognition was measured using the facial expression recognition paradigm from the emotion understanding measurement task, which included two parts: expression naming and expression identification (Pons et al., 2002). In the expression naming task, the experimenter individually presented four pictures depicting happy, sad, angry, and fearful expressions to each child (the gender and age of the characters in the pictures were consistent with those of the child), and the child was asked to name each of the four expressions. Two points were awarded for a correct naming and zero points for an incorrect naming. In the expression identification task, the child was asked to identify the target expression from among four pictures depicting happy, sad, angry, and fearful expressions presented simultaneously. One point was awarded for a correct identification and zero points for an incorrect identification. The facial expression recognition score was obtained by adding the scores from the expression naming and expression identification tasks. The cartoon face pictures used in this task were drawn by a graduate student majoring in preschool education with excellent drawing skills. The recognition rate of facial expressions was tested by 30 college students (15 males and 15 females), and eight pictures with a recognition rate of 100% were selected. The research materials are shown in Figure 2.

#### 2.2.3 Emotion situation understanding

The emotion situation understanding paradigm based on external factors, desires, beliefs, and cues was adapted from the emotion understanding measurement task (Wang et al., 2019). Each task used a story-based method (with corresponding storybooks provided to facilitate children’s understanding), with each independent story representing a level of emotion understanding task. To avoid difficulties in recognition and judgment caused by age and gender, the characters in the pictures were similar in age to the participants, and both male and female versions of the pictures and comics were included. While presenting the emotional story pictures to the children, the experimenter used a neutral tone and expression to tell the corresponding story to the children. Then, the children were asked to judge the emotional state of the story character based on the situational information (by identifying from the given four pictures of facial expressions). In the emotion entry section, the character’s facial expression was left blank, and the emotion option section presented four emotion faces of happy, sad, angry, and fearful. One point was awarded for a correct selection and zero points for an incorrect selection. The emotion understanding tasks based on external factors and desires each contained four questions, with a total score of four points. The emotion understanding tasks based on beliefs and cues each contained a control question (not scored, but incorrect answers needed to be corrected) and a target question (one point for a correct answer and zero points for an incorrect answer), with a total score of one point. Experts in the field of preschool education and developmental psychology were invited to discuss and determine the selected stories for each task, which were in line with children’s life experiences, easy for children to understand, and relevant to the research topic. The detailed tasks are shown in Table 1.

**Table 1:** Emotion situation understanding tasks selected for this study.

Emotion understanding measurement task (TEC) (From the beginning of each task, one point is awarded for each correct answer and zero points for incorrect answers. The results are calculated based on the average score)					
Emotion understanding based on external factors	Question		Answer	Correct answer	Score
	Turtle: A little boy sees his pet turtle has died. How does he feel now?			Sad	
	Gift: A little boy receives a gift on his birthday. How does he feel now?			Happy	
	Disturbance: A little boy is doing his homework when his little brother comes and disturbs him. How does he feel now?			Angry	
	Lion: A little boy is chased by a fierce lion. How does he feel now?			Scared	
Emotion understanding based on desires	Question				
	1. A little boy likes pears, and a little girl does not like them. Can you tell the teacher who likes pears? (Incorrect answers need to be corrected.)		2. A little boy does not like cake, and a little girl likes it. Can you tell the teacher who likes cake? (Incorrect answers need to be corrected.)		
	(1) A little boy opens a box and sees pears inside. How does he feel now? (Happy)	(2) A little girl opens a box and sees pears inside. How does she feel now? (Angry/Sad)	(1) A little boy opens a box and sees cake inside. How does he feel now? (Angry/Sad)	(2) A little girl opens a box and sees cake inside. How does she feel now? (Happy)	
	Answer and score		Answer and score		
Emotion understanding based on beliefs	Question			Answer	
	Xiao Ming has planted some plants in the yard. One morning, he went to the yard and saw that all his plants had bloomed beautifully. However, just after he left, a big dog ran into the yard and trampled on all his flowers. Can you tell the teacher if Xiao Ming knows that his flowers have been trampled on? (He does not know. Incorrect answers need to be corrected: Xiao Ming does not know that his flowers have been trampled on.)				
	Xiao Ming does not know that his flowers have been trampled on. How does he feel now? (Answer: Happy/Excited)				
Emotion understanding based on cues	Question			Answer	
	Xiao Ming is angry because he saw his flowers trampled by the big dog. A few days later, Xiao Ming is playing hide-and-seek with his good friend in the park.				
	Control Question: Xiao Ming and his friends are playing their favorite game of hide-and-seek. How does he feel now? (Answer: Happy, incorrect answers need to be corrected)				
	Target Question: While they are playing, Xiao Ming suddenly sees a big dog that looks exactly like the one that trampled his flowers. How does he feel now? (Answer: Angry)				

### 2.3 Research process

Firstly, we communicated the entire research design with the target kindergarten teachers and made modifications based on their feedback. The modifications primarily focused on aspects such as the timing and logistics of conducting the tests, as well as any specific considerations related to the kindergarten’s educational context. These modifications did not alter the fundamental research objectives or the measures used in the study. Secondly, we trained the experimenters and conducted a pilot test. All experimenters who participated in this study were psychology graduate students who had received systematic training. The training encompassed detailed instructions on administering the cognitive flexibility and emotion understanding tasks, ensuring consistent and standardized tasks, ensuring consistent and standardized administration across all participants. Then, we selected the participants and conducted the tests. Each preschool child participant was required to complete the cognitive flexibility and emotion understanding tasks in a quiet, bright, and tidy classroom. We conducted individual tests during the children’s free play time. After completing all the tasks, the children received a beautiful cartoon sticker as a gift. Finally, we reported the research results to the preschool children’s parents and teachers during a parent-teacher meeting. We shared the research findings, discussed their implications, and addressed any questions or concerns raised by the parents and teachers.

### 2.4 Statistical analysis

In this study, we used SPSS 23.0 statistical software to conduct the following statistical tests: Descriptive statistics were used to summarize and describe the key characteristics, measures such as means, standard deviations, and frequencies were computed to provide an overview of the data. ANOVA allowed us to explore potential within-subject differences and interactions between the age and gender. Pearson correlation was conducted to examine the relationships between cognitive flexibility and emotion understanding. Regression analysis was performed to investigate the predictive of cognitive flexibility on emotion understanding.

## 3 Results

### 3.1 Analysis of cognitive flexibility characteristics in 3–6-year-old children

The results indicated that overall, preschoolers showed a certain level of development In cognitive flexibility, facial expression recognition, and emotion understanding, But with individual differences. Cognitive flexibility demonstrated significant differences across different Age groups and genders, while facial expression recognition and emotion-scenario understanding also varied across emotional conditions and Age groups. Furthermore, regression analysis revealed a significant positive relationship between cognitive flexibility and emotion understanding, even when controlling for Age and gender.

Descriptive statistics were performed on the average cognitive flexibility scores of preschool children. The overall average score was 0.919, with scores ranging from 0.28 to 1 (full score being 1). This indicates that the overall cognitive flexibility development of preschool children is at a relatively high level. A two-factor ANOVA was conducted on the average cognitive flexibility scores across different age groups (3–3.5 years, 3.5–4 years, 4–4.5 years, 4.5–5 years, 5–5.5 years, 5.5–6 years, 6–6.5 years) and genders (male, female). The results showed significant differences in cognitive flexibility across different age groups [*F*_(6,517)_ = 12.797, *p* < 0.001, *η*^2^ = 0.097]. Further analysis showed that children under 5 years old had lower cognitive flexibility scores compared to those over 5 years old. Specifically, the order was 3–3.5 years (0.813 ± 0.186) > 3.5–4 years (0.848 ± 0.212) > 4–4.5 years (0.921 ± 0.102) > 4.5–5 years (0.922 ± 0.106) > 5–5.5 years (0.957 ± 0.086) > 6–6.5 years (0.967 ± 0.064) > 5.5–6 years (0.58 ± 0.080). There was also a significant difference in cognitive flexibility between genders [*F*_(1,509)_ = 19.284, *p* < 0.001, *η*^2^ = 0.102], with females (0.938 ± 0.008) scoring significantly higher than males (0.888 ± 0.008) (see Table 2 for details).

**Table 2:** Analysis of variance results for cognitive flexibility.

Age									Gender			Total
Cognitive flexibility	3 ~ 3.5	3.5 ~ 4	4 ~ 4.5	4.5 ~ 5	5 ~ 5.5	5.5 ~ 6	6 ~ 6.5	F	Male	Female	F	
	0.813 ± 0.186	0.848 ± 0.212	0.921 ± 0.102	0.922 ± 0.106	0.957 ± 0.086	0.967 ± 0.064	0.958 ± 0.080	19.284^***^	0.888 ± 0.008	0.938 ± 0.008	19.284^***^	0.919 ± 0.131

***p < 0.001.

### 3.2 Analysis of facial expression recognition characteristic in 3–6-year-old children

Descriptive statistics were performed on the overall average score of facial expression recognition in preschoolers. The mean score was 1.695, with scores ranging from 0.5 to 2 (full score being 2). This indicates that the overall ability of facial expression recognition in preschool children is at a moderately high level, with large individual differences. A 4 (condition: happy, sad, angry, scared) × 2 (gender: male, female) × 7 (age: 3–3.5 years, 3.5–4 years, 4–4.5 years, 4.5–5 years, 5–5.5 years, 5.5–6 years, 6–6.5 years) three-factor repeated measures ANOVA was conducted, with gender and age as between-subject variables, different emotional conditions as within-subject variable, and facial expression recognition mean score as dependent variable. The results showed a significant main effect of condition [*F*_(3, 1,521)_ = 234.217, *p* < 0.001, *η*^2^ = 0.316]. *Post hoc* tests indicated that children’s recognition of angry (1.904 ± 0.019), happy (1.943 ± 0.013), and sad (1.858 ± 0.024) expressions was higher than that of scared expressions (1.055 ± 0.047). The main effect of age was also significant [*F*_(6, 507)_ = 8.846, *p* < 0.001, *η*^2^ = 0.095]. *Post hoc* tests showed that the facial expression recognition scores were ordered as 3–3.5 years (1.474 ± 0.049) < 3.5–4 years (1.581 ± 0.040) < 4–4.5 years (1.653 ± 0.030) < 4.5–5 years (1.695 ± 0.037) < 5–5.5 years (1.816 ± 0.035) < 6–6.5 years (1.774 ± 0.039) < 5.5–6 years (1.837 ± 0.054). There was a significant main effect of gender [*F*_(1, 507)_ = 6.311, *p* = 0.012, *η*^2^ = 0.012]. *Post hoc* tests showed that girls (1.729 ± 0.023) had higher emotion recognition scores than boys (1.651 ± 0.021). The interaction between condition and gender was marginally significant [*F*_(3, 11,521)_ = 2.357, *p* = 0.070, *η*^2^ = 0.005]. Simple effects analysis showed no significant gender differences in scores for angry, happy and sad expressions, *p*s > 0.05, but for scared expressions, girls (1.158 ± 0.068) scored higher than boys (0.952 ± 0.063). See Table 3 for descriptive statistics and ANOVA results of facial expression recognition scores across conditions, age groups and genders.

**Table 3:** Analysis of variance results for facial expression recognition and emotional contextual understanding in preschoolers across condition, age and gender.

		Facial expression recognition					Emotional contextual understanding				
		Angry	Scare	Happy	Sad	*F*	Feelings	Desires	Beliefs	Cues	*F*
Age	3 ~ 3.5	1.605 ± 0.059	0.726 ± 0.147	1.799 ± 0.042	1.764 ± 0.076	1.509	0.831 ± 0.026	0.824 ± 0.025	0.052 ± 0.053	0.440 ± 0.068	2.779^***^
	3.5 ~ 4	1.912 ± 0.048	0.882 ± 0.119	1.853 ± 0.034	1.676 ± 0.062		0.849 ± 0.021	0.875 ± 0.020	0.044 ± 0.043	0.588 ± 0.055	
	4 ~ 4.5	1.899 ± 0.036	0.980 ± 0.090	2.000 ± 0.026	1.732 ± 0.046		0.886 ± 0.016	0.920 ± 0.015	0.098 ± 0.032	0.604 ± 0.041	
	4.5 ~ 5	1.950 ± 0.044	0.999 ± 0.110	1.950 ± 0.031	1.882 ± 0.057		0.895 ± 0.019	0.956 ± 0.019	0.126 ± 0.039	0.557 ± 0.050	
	5 ~ 5.5	1.981 ± 0.043	1.309 ± 0.106	2.000 ± 0.030	1.972 ± 0.055		0.951 ± 0.019	0.976 ± 0.018	0.236 ± 0.038	0.838 ± 0.048	
	5.5 ~ 6	1.977 ± 0.046	1.143 ± 1.116	2.000 ± 0.033	1.977 ± 0.060		0.947 ± 0.020	0.981 ± 0.020	0.296 ± 0.041	0.829 ± 0.053	
	6 ~ 6.5	2.000 ± 0.064	1.347 ± 0.161	2.000 ± 0.046	2.000 ± 0.083		0.987 ± 0.028	0.990 ± 0.028	0.360 ± 0.058	0.920 ± 0.074	
Gender	Male	1.891 ± 0.025	0.952 ± 0.063	1.921 ± 0.018	1.838 ± 0.033	2.357	0.902 ± 0.011	0.932 ± 0.011	0.131 ± 0.023	0.688 ± 0.029	2.287
	Female	1.916 ± 0.027	1.158 ± 0.068	1.965 ± 0.020	1.877 ± 0.035		0.911 ± 0.012	0.932 ± 0.012	0.215 ± 0.024	0.677 ± 0.031	
Condition		1.904 ± 0.019	1.055 ± 0.047	1.943 ± 0.013	1.858 ± 0.024	234.217^***^	0.907 ± 0.008	0.932 ± 0.008	0.173 ± 0.017	0.682 ± 0.021	611.400^***^

***p < 0.001.

### 3.3 Analysis of variance in emotion-scenario understanding

Descriptive statistics were performed on the overall average score of preschoolers’ emotion-scenario understanding. The mean score was 0.668, with scores ranging from 0.06 to 1 (full score being 1). This indicates that the development of emotion-scenario understanding in preschool children is still immature, with large individual differences. A 4 (condition: emotion understanding based on external causes, desires, beliefs, and cues) × 2 (gender: male, female) × 7 (age: 3–3.5 years, 3.5–4 years, 4–4.5 years, 4.5–5 years, 5–5.5 years, 5.5–6 years, 6–6.5 years) three-factor repeated measures ANOVA was conducted, with gender and age as between-subject variables, different emotion conditions as within-subject variable, and emotion-scenario understanding mean score as dependent variable.

The results showed a significant main effect of condition [*F*_(3, 1,536)_ = 611.400, *p* < 0.001, *η*^2^ = 0.544]. *Post hoc* tests indicated that scores for emotion understanding based on external causes (0.907 ± 0.008) and desires (0.932 ± 0.008) were significantly higher than those based on beliefs (0.173 ± 0.017) and cues (0.682 ± 0.021). The main effect of age was also significant [*F*_(6, 512)_ = 20.606, *p* < 0.001, *η*^2^ = 0.195]. *Post-hoc* tests showed that emotion-scenario understanding scores were ordered as 3–3.5 years (0.537 ± 0.025) < 3.5–4 years (0.589 ± 0.021) < 4–4.5 years (0.627 ± 0.015) < 4.5–5 years (0.634 ± 0.019) < 5–5.5 years (0.750 ± 0.018) < 5.5–6 years (0.763 ± 0.020) < 6–6.5 years (0.814 ± 0.028). The interaction between condition and age was significant [*F*_(18, 1,536)_ = 2.779, *p* = 0.001, *η*^2^ = 0.032]. Simple effects analysis showed no significant differences across age groups for external cause- and desire-based emotion understanding, *p*s > 0.05, but significant differences across age groups for belief- and cue-based emotion understanding, *p*s < 0.05. See Table 3 for descriptive statistics and ANOVA results of emotion-scenario understanding scores across conditions, age groups and genders.

### 3.4 An analysis of the relationship between cognitive flexibility and emotion understanding in preschool children

#### 3.4.1 Correlational analysis of cognitive flexibility and different components of emotion understanding

First, we examined the Pearson correlation among cognitive flexibility, facial expression recognition, externally caused emotion understanding, desire-based emotion understanding, belief-based emotion understanding, and cue-based emotion understanding. The results showed that except for the non-significant correlation between emotion recognition and cue-based emotion understanding, all other variables were significantly positively correlated with each other. The results of the correlational analysis are presented in Table 4.

**Table 4:** Analysis of the correlations between cognitive flexibility and the dimensions of emotional understanding.

	1	2	3	4	5	6
1 Cognitive flexibility	1					
2 Emotion face recognition	0.164**	1				
3 Externally-based emotion understanding	0.244**	0.234**	1			
4 Desire-based emotion understanding	0.234**	0.144**	0.254**	1		
5 Belief-based emotion understanding	0.181**	0.104**	0.084*	0.103**	1	
6 Cue-based emotion understanding	0.096^**^	0.041	0.230**	0.141*	0.095*	1

*p < 0.05. **p < 0.01.

#### 3.4.2 Regression analysis of preschoolers’ cognitive flexibility and the dimensions of emotion understanding

The previous analysis found that both age and gender influence preschoolers’ cognitive flexibility and emotional understanding. To further examine the relationship between cognitive flexibility and emotional understanding, regression analysis was conducted with cognitive flexibility as the predictive variable and preschoolers’ scores on each emotional understanding task as outcome variables, after controlling for age and gender. The results showed that cognitive flexibility significantly and positively predicted emotion face recognition (*β* = 0.271, *p* = 0.026), externally-based emotion understanding (*β* = 0.261, *p* < 0.001), and desire-based emotion understanding (*β* = 0.261, *p* = 0.001). See Table 5 for the regression analysis results.

**Table 5:** Regression analysis of cognitive flexibility on emotional understanding tasks.

	*β*	*t*	*p*	*R*^2^
Emotion face recognition	0.271	2.230	0.026	0.081
Externally-based emotion understanding	0.261	4.399	< 0.001	0.099
Desire-based emotion understanding	0.198	3.397	0.001	0.090
Belief-based emotion understanding	0.184	1.498	0.135	0.085
Cue-based emotion understanding	0.183	1.157	0.248	0.081

## 4 Discussion

In summary, the study sheds light on the cognitive and emotional development of preschoolers aged 3–6. Cognitive flexibility, emotion understanding were key focus areas. The DCCS task and emotion understanding tasks were used to investigate the developmental characteristics of cognitive flexibility.

### 4.1 Discussion on cognitive flexibility in 3–6-year-old preschoolers

This study found that preschoolers’ accuracy rate on the cartoon sorting task reached over 90%, indicating that cognitive flexibility of preschoolers has overall reached a relatively high level. This is basically consistent with the findings of a recent study (Wang et al., 2019). However, some other studies have drawn inconsistent conclusions (Peng et al., 2017), which may be related to the heterogeneity of experimental tasks and the timing of experiment implementation. The DCCS task was specially developed for preschool children, and with the advancement of social development and earlier maturation of preschoolers’ cognitive abilities, the DCCS paradigm can easily lead to a ceiling effect. In addition, this study also found that there were significant differences in preschoolers’ cognitive flexibility at different age stages, showing an increasing trend with age, with a major improvement around 5 years old, approaching a perfect score. This suggests that as knowledge increases and cortical development progresses, preschoolers’ ability to flexibly switch between two incompatible rule sets is gradually enhanced, and cognitive flexibility undergoes a qualitative leap at age 5. This study also found that girls’ cognitive flexibility was significantly higher than boys’, which may be related to girls being relatively better at following instructions, and having relatively better attentiveness and memory compared to boys.

### 4.2 Discussion on facial expression recognition in 3–6-year-old preschoolers

First, we found that preschoolers’ recognition accuracy for fearful expressions was significantly lower than for angry, happy, and sad expressions. The reasons may be related to preschoolers’ life experiences. Specifically, preschoolers experience many events in their growth that make them feel happy, sad, and angry, while under parents’ protection, preschoolers have relatively fewer experiences of fear emotions, so they are relatively unfamiliar with such emotional experiences and have relatively poorer ability to identify corresponding expressions. Second, we found that the developmental trends for recognizing happy, sad, and angry facial expressions were basically consistent, with only a few 3-year-olds making recognition errors. However, only very few 3-year-olds could recognize fearful expressions, and by the end of preschool, only about half of 6-year-olds could recognize this expression. This is consistent with the findings of Mou Lixia et al., indicating preschoolers’ recognition of happy, sad, and angry expressions is relatively mature, while the ability to recognize fearful expressions may not fully develop until school age. Finally, we also found that girls’ overall facial expression recognition ability was slightly better than boys’. This is consistent with most domestic and foreign research results (Li et al., 2016; Fidalgo et al., 2018). Scholars believe this phenomenon may be related to parents’ greater attention to girls’ emotions, causing girls to be more sensitive in perceiving emotions and better at inferring emotional outcomes from facial expression cues (Liang et al., 2011). This difference mainly manifests in recognizing fearful expressions. We believe that under normal circumstances, male preschoolers have better psychological resilience than females, so they do not express fear as strongly as female preschoolers, leading to less sensitivity in recognizing fearful expressions.

### 4.3 Discussion on emotion and situation understanding of children aged 3–6

First, we found that preschool children have the strongest ability to understand emotions based on external causes and wishes, followed by emotion understanding based on cues, and the weakest ability in emotion understanding based on beliefs. This may be because beliefs are more complex in different mental states, as they involve representations or explanations of the world, and thus are relatively difficult to understand, while some other mental states, such as wishes and intentions, can be explained non-representatively, and thus are easier to understand (Li and Su, 2004). Due to the different levels of difficulty in understanding these mental states, these four types of emotion situation understanding abilities also show specificity in developmental order. Second, we also found that the scores for emotion understanding based on external causes and wishes remained relatively stable during the preschool period, while the scores for emotion understanding based on beliefs and cues increased with age, which may be related to the development of children’s theory of mind. According to Piaget’s cognitive development theory, three-year-olds are context-dependent, able to project their own experiences in situations to others; four-year-olds are in the stage of social knowledge dependence, able to make stereotyped inferences based on existing social knowledge (Huitt and Hummel, 2003). Correspondingly, three-year-olds can evaluate the causes of emotions and it is not difficult to understand the emotional states when wishes are unfulfilled or satisfied (Dunn, 2010). Emotion understanding based on cues/memory requires preschoolers to make flexible inferences based on specific behavioral information, and 5-year-olds are in the behavior information dependent stage, so emotion understanding based on cues develops rapidly at this stage. While for emotion understanding based on beliefs, children need to make comprehensive inferences based on situational information, existing knowledge and specific object behaviors, which ability generally develops at around 6 years old when children enter the stage of integration of information. Therefore, we speculate that emotion understanding based on beliefs can develop rapidly in the late preschool and school-age periods. Finally, we found that there were no significant gender differences in emotion understanding based on external causes, wishes and cues, while girls scored higher than boys in emotion understanding based on beliefs, which shows that simple emotion situation understanding as a basic emotional ability has little gender difference, while in the process of socializing preschool children, gender differences have emerged in some relatively complex emotion understanding tasks, showing that girls perform better than boys.

### 4.4 Discussion on the relationship between cognitive flexibility and emotion understanding in children aged 3–6

Cognitive flexibility and emotion understanding are two very important parts of children’s social cognition. This study found that there were significant correlations between children’s cognitive flexibility tasks and emotion understanding tasks, even after controlling for age and gender, cognitive flexibility still predicted the first three dimensions of emotion understanding. One interpretation of the above findings is that cognitive flexibility enables children to overcome perceptual salience by coordinating the transformation between external behavior and internal cues to identify and interpret their own and others’ emotions. Children with higher cognitive flexibility are more likely to recognize that the same behavior can cause different or even opposite feelings in different situations, so they can make more accurate judgments, which indicates that cognitive flexibility may be a basic guaranteeing ability for emotion understanding ability (Li et al., 2019a,b; Wang et al., 2021). After eliminating the factors of age and gender, cognitive flexibility no longer has predictive power for emotion understanding based on beliefs and emotion understanding based on cues, on the one hand, it may be because preschool children’s development in these two aspects is generally low; on the other hand, it may also be related to the DCCS task used in this study to measure children’s cognitive flexibility, this task only includes children’s basic cognitive ability to switch rules in color and shape two dimensions, while for emotion understanding involving higher-order cognitive abilities including beliefs and cues, a more refined cognitive flexibility measurement task may be needed to predict.

## 5 Educational implications

### 5.1 Parents and kindergarten educators should pay attention to the cultivation of children’s cognitive flexibility and provide personalized support

The cultivation of cognitive flexibility in preschoolers should attract the attention of early educators, though most preschoolers develop cognitive flexibility at a high level. Some preschoolers have delayed development of cognitive flexibility, and those preschoolers should be given the most support. Spiro’s Cognitive Flexibility Theory posits that teachers should help students construct their own knowledge to help children make appropriate responses in complex situations (Spiro, 1988). Specifically, kindergarten teachers should pay attention to guiding preschoolers’ multi-dimensional representation of things in daily activities and group teaching; frequently remind preschoolers of other ways to solve problems, help preschoolers change rigid thinking, promote their flexible conversion and application of knowledge; Finally, kindergarten teachers should pay more attention to preschoolers, identify those with slow development of cognitive flexibility in time, and strengthen “personalized” support for preschoolers at different development levels.

### 5.2 Parents and early educators should provide targeted cultivation of preschoolers’ emotion understanding abilities based on an accurate grasp of the characteristics of preschoolers’ emotion understanding development

First, in daily life and teaching, parents and early educators may need to pay more attention to the development of children’s fear emotions. Parents and teachers should apply various means in an integrated way to guide and help children understand and express this emotion. Guide children to understand that fear is a normal psychological emotion; then through interpreting the content of fear, cultivate children’s ability to identify this expression in themselves and others; so as to cultivate children’s proper responses to this emotion, in order to protect children from harm in appropriate situations. Parents and teachers should strengthen the cultivation of children’s understanding of beliefs and application of cues. In daily life, use more psychological terms such as hope, think, feel and intend, through establishing high-quality conversations and interactions with children, guide children to think from multiple perspectives, in order to promote children’s understanding of their own and others’ emotional states.

Next, parents and teachers should pay attention to the development of children’s emotion understanding abilities at different levels in the corresponding age stages. Parents and teachers need to grasp the 5-year-old developmental turning point in time. Once it is found that children’s development is poor in certain aspects, certain training should be provided to improve it. At the same time, this insight also reminds us that while cultivating children’s emotion understanding abilities, we should also consider the current development level of preschoolers at different age stages. In choosing emotion cognition learning materials, we should consider the overall difference in acceptability of preschoolers at different age stages to ensure that the corresponding emotion education measures can be implemented smoothly.

Finally, in daily education, more attention should be paid to children’s (especially boys) emotions. For parents, they should actively take on the role of creating a positive family emotional atmosphere, interact more with children at the emotional level, and actively and proactively use parent–child emotion-themed picture books and parent–child emotion communication skills to help children improve their emotion understanding abilities. For early educators, they should pay more attention to children’s emotional reactions, communicate more with children about their emotional feelings at the moment, guide children to learn to accurately express their emotions with language, and root emotion education in children’s daily lives.

### 5.3 Parents and early educators should make good use of cognitive flexibility training to improve preschoolers’ levels of emotion understanding

Cognitive flexibility is closely related to preschoolers’ emotion understanding abilities, and it may be a basic guaranteeing ability for children’s emotion understanding. Cognitive flexibility training can help preschoolers control their advantage responses in complex situations, flexibly approach problems from different angles, and thus improve their emotion understanding abilities. Therefore, teachers can try using some games that involve cognitive flexibility elements to improve preschoolers’ emotion understanding abilities, common types of games include task switching games and role-playing games. In task switching games, there are usually multiple game rules, and after children are familiar with the original rules, the game rules are changed, which requires preschoolers to inhibit advantage responses when answering and switch to rule execution responses that meet the current task requirements; In role-playing games, preschoolers need to inhibit their personality performance, remember their own and other children’s roles, flexibly respond to other children’s behavior performance, which helps to improve executive functions such as inhibition control, working memory and cognitive flexibility. In addition, Li Quan and others found that mindfulness training can promote the development of children’s cognitive flexibility (Li et al., 2019a,b). This suggests that kindergarten teachers can incorporate mindfulness training into preschoolers’ daily activities to guide preschoolers to focus their attention on the present experience, improve preschoolers’ cognitive flexibility, and thus improve preschoolers’ emotion understanding abilities.

## Data availability statement

The raw data supporting the conclusions of this article will be made available by the author, without undue reservation.

## Ethics statement

Ethical review and approval was not required for the study on human participants in accordance with the local legislation and institutional requirements. Written informed consent to participate in this study was provided by the participants’ legal guardian/next of kin.

## Author contributions

LM: Conceptualization, Formal analysis, Investigation, Methodology, Writing – original draft.
